# A Rapid NGS-Based Preimplantation Genetic Testing for Chromosomal Abnormalities in Day-3 Blastomere Biopsy Allows Embryo Transfer Within the Same Treatment Cycle

**DOI:** 10.3389/fgene.2021.636370

**Published:** 2021-02-26

**Authors:** Yinghui Ye, Jieliang Ma, Long Cui, Sijia Lu, Fan Jin

**Affiliations:** ^1^Key Laboratory of Reproductive Genetics (Ministry of Education), Department of Reproductive Endocrinology, Women’s Hospital, Zhejiang University School of Medicine, Hangzhou, China; ^2^Department of Clinical Research, Yikon Genomics Co. Ltd., Suzhou, China; ^3^Women’s Reproductive Health Laboratory of Zhejiang Province, Women’s Hospital, Zhejiang University School of Medicine, Hangzhou, China

**Keywords:** preimplantation genetic testing, next-generation sequencing, chromosomal rearrangements, blastomere biopsy, aneuploidy

## Abstract

Nowadays, most of the preimplantation genetic testing (PGT) is performed with a strategy of comprehensive chromosome screening and trophectoderm biopsy. Nevertheless, patients with ovarian insufficiency may not have competent blastocysts. In the present study, we aimed to establish the value of multiple annealing and looping-based amplification cycle (MALBAC)-based next-generation sequencing (NGS) for PGT in day-3 embryos. A total of 94.3% (1168/1239) of embryos yielded informative results, and the overall embryo euploid rate was 21.9% (256/1168). Overall, 225 embryos were transferred in 169 cycles with a clinical pregnancy rate of 49.1% (83/169). The live birth and implantation rates were 47.3% (80/169) and 44.4% (100/225), respectively. Double embryos transfer showed higher clinical pregnancy and live birth rates compared with single embryo transfer, but the implantation rates were similar (44.2% vs. 44.6%, *P* > 0.05). The euploid rate for reciprocal translocations (16.1%) was significantly lower than that for Robertsonian translocations (28.0%, *P* < 0.01) and inversions (28.0%, *P* < 0.01). However, higher percentages of embryos with *de novo* abnormalities were observed with Robertsonian translocations (23.3%, *P* < 0.01) and inversions (30.5%, *P* < 0.01) than with reciprocal translocations (11.6%). We demonstrated that NGS for PGT on day-3 embryos is an effective clinical application, particularly for patients with a diminished ovarian reserve and limited embryos.

## Introduction

Preimplantation genetic testing for aneuploidy (PGT-A) and preimplantation genetic testing for chromosomal structural rearrangements (PGT-SR) are effective approaches for selecting euploid embryos for transfer to lower the risks of implantation failure and miscarriage in the clinical treatment of *in vitro* fertilization (IVF) (Munné et al., 2006; Hodes-Wertz et al., 2012; Forman et al., 2013; Christodoulou et al., 2017). Initially, fluorescence *in situ* hybridization (FISH) was used for single blastomere PGT at the cleavage stage, although it can only examine 5–10 unique chromosomes. Furthermore, several randomized controlled trials verified that FISH cannot truly improve IVF outcomes (Mastenbroek et al., 2011; Rubio et al., 2013) since *de novo* abnormalities may be ignored if they occur in any undetected chromosomes (Ye et al., 2011). Another aspect related to unfavorable PGT results is that cleavage-stage embryos have a higher chance of being mosaic for aneuploidies (Vanneste et al., 2009; Mertzanidou et al., 2013); therefore, the PGT results may not be representative of the genuine genetic status of the embryos. Currently, the majority of PGT is performed with a strategy of genetic testing with comprehensive chromosome screening (CCS) methods to evaluate the genetic abnormalities of all 23 chromosome pairs and trophectoderm (TE) biopsy at the blastocyst stage (Coonen et al., 2020). All CCS methods, including array comparative genomic hybridization (aCGH), single-nucleotide polymorphism (SNP) array, and next-generation sequencing (NGS) technology, have been verified to effectively improve PGT outcomes by detecting aneuploidy and segmental aneuploidy (Brezina et al., 2016). However, despite improvements in culture conditions and laboratory techniques, only approximately 50% of human embryos develop to the blastocyst stage *in vitro* (McCollin et al., 2020). Besides, patients with insufficient ovarian function may not have sufficient embryos for PGT. Thus, embryo biopsy at the cleavage stage would be an alternative solution for these patients. Although cleavage-stage PGT by FISH has failed to demonstrate beneficial results, encouraging clinical outcomes of CCS-based PGT-A following day-3 embryo biopsy compared with routine IVF have been observed in some studies (Keltz et al., 2013; Lukaszuk et al., 2015). However, there is still a lack of large-scale clinical data to fully verify these outcomes.

For carriers of balanced structural chromosomal rearrangement, such as reciprocal translocations, Robertsonian translocations, and inversions, most of the unbalanced gametes produced are considered to be the result of different segregation events in translocation carriers or recombination events in inversion carriers and present aneuploidy for chromosomes involved in the rearrangements. However, previous CCS-based PGT-SR studies reported that some of the chromosomal abnormalities detected in embryos were not related to parental rearrangements (Alfarawati et al., 2012; Mateu-Brull et al., 2019). Several studies ascribe a portion of the *de novo* aneuploidies to the inter chromosomal effect (ICE) (Lejeune, 1963): chromosomes involved in rearrangements may interfere with the segregation of other chromosomes by disrupting chromosome alignment on the spindle during meiosis (Wang et al., 2019). However, with different studies presenting contradictory results, the existence of ICE remains controversial.

In this study, we evaluated the applicability of a novel NGS-PGT strategy based on an improved multiple annealing and looping-based amplification cycle (MALBAC) whole-genome amplification (WGA) for cleavage-stage embryos in detecting aneuploidies and unbalanced translocation/inversion products. The good clinical outcomes of the present study demonstrated the value of NGS on day-3 embryo biopsy for PGT of chromosomal abnormalities. We also analyzed the incidence of parental rearrangement-related and *de novo* chromosomal abnormalities in embryos from carriers with different types of structural rearrangements.

## Materials and Methods

### Study Population

The study was performed at the Department of Reproductive Endocrinology, Women’s Hospital, Zhejiang University School of Medicine. The current study was approved by the Medical Ethics Committee of the Women’s Hospital, Zhejiang University School of Medicine (IRB-20200242-R). A total of 283 patients underwent 329 cycles of IVF with single blastomere PGT using a ChromInst Library Preparation Kit (Yikon Genomics, Cat. no XK008) between 2016 and 2018. Among all the cycles, 171 had reciprocal translocations, 77 had Robertsonian translocations, 34 had inversions, 3 had insertions, 2 had fragment duplications (1 patient: 46,Y,dup(X)(q21.31q22.1), SNP array: Xq21.31q22.1 duplication, 13.4 Mb), 3 had microdeletions(2 patients: case 1: 46,XX,del(21), SNP array: 21q22.3 deletion, 4.6 Mb; case 2: 46,X,del(X)(p22.2p22.33), SNP array: Xp22.33p22.2 deletion, 16.0 Mb), and 41 cycles were free of chromosomal rearrangements (including Y chromosome microdeletions and sex chromosome abnormalities).

### Stimulation Protocol

All cycles were subjected to controlled ovarian hyperstimulation protocols. Ovarian stimulation was performed for patients with normal ovarian function using the standard long-term GnRH agonist protocol. For patients with poor ovarian reserves (follicle-stimulating hormone level > 12 mIU/mL or antral follicle count < 5), a GnRH antagonist protocol or mild stimulation was used. Transvaginal sonography and serial E2 levels were used to monitor ovarian follicular development. Once a dominant follicle reached 19–20 mm, 10,000 IU of hCG was given. Thirty-six hours later, transvaginal ultrasound-guided oocyte retrieval was performed.

### Fertilization, Culture, and Embryo Biopsy

Insemination was performed with intracytoplasmic sperm injection (ICSI) for all cycles. Fertilization was confirmed 17–20 h later with the presence of two pronuclei (2PN). Normally fertilized 2PN zygotes were transferred into G-1 medium (Vitrolife, Sweden) until day 3 of embryo development. Single blastomere biopsy of good-quality embryos (defined as follows: 6–10 cells, < 25% fragmentation, equally sized mononucleated blastomeres) was performed on day 3 of the culture. In cycles not suitable for fresh embryo transfer, good-quality embryos were cryopreserved by slow-freezing methods using FreezeKit Cleave^TM^ (Vitrolife, Sweden). After thawing, the embryos were cultured in G-1 medium for 2 h before the biopsy. Embryos underwent biopsy individually in G-PGD medium (Vitrolife, Sweden). The criteria for selection of blastomeres were the presence of a nucleus and maintenance of cell integrity. After the biopsy, the embryos were washed and transferred to G2 medium (Vitrolife, Sweden). The biopsied blastomeres were immediately washed in 1% polyvinylpyrrolidone and transferred into sample collection tubes (Yikon Genomics, Cat. no. XK-043).

### Whole-Genome Amplification and Next-Generation Sequencing

An improved MALBAC WGA strategy was used for this study. Blastomeres were subjected to lysis and WGA following the instructions of a ChromInst Library Preparation Kit (Yikon Genomics, Cat. no XK-008), which combines WGA pre-amplification (Pre-AMP) with library construction in only one step. Random primers (Pre-AMP primer) with universal sequences are first annealed to genomic DNA molecules and are then extended by DNA polymerase. Universal regions in the Pre-AMP primer enable initial full amplicons to form loops, thereby excluding them as templates for further amplification. After several cycles of linear Pre-AMP, only full amplicons can be exponentially amplified during PCR with AMP primers 1 and 2. The AMP primers have complementary regions that are designed to anneal to the full amplicons. The additional linked sequences of the AMP primers are designed to introduce P7 and P5 primers for the Illumina sequencer platform. Through this design, a sequencing-ready library is prepared in one amplification step. The entire process can be accomplished in about 2.5 h.

The concentration of the libraries was quantified by a Qubit 3.0 Fluorometer (Life Technologies). We performed sequencing with single-end read 55 bp on the Illumina Nextseq 550 platform. A total of ∼3 million valid reads were obtained per sample, which yielded an average 4% genome coverage. Subsequently, the read numbers were counted along the whole genome with a bin size of 1 Mb and normalized by the GC content and a reference dataset. A copy number gain of two to three copies results in a 50% increase in read counts, whereas a copy number loss of two copies to one copy results in a 50% decrease in read counts. Therefore, a 0.04 × genome-wide depth was obtained to analyze the copy number variations up to 1 Mb resolution (Huang et al., 2014). Embryos showed > 40% mosaic rate would be indicated as mosaic aneuploidy and not fit for transfer.

### Transfer Strategy

Embryo transfer was performed on the next day following embryo biopsy. One or two euploid embryos were transferred after evaluation of the embryo quality. We generally recommended transferring one embryo, but patients could choose to transfer one or two embryos if they had two or more euploid embryos. Surplus embryos were cryopreserved.

### Statistical Analysis

Clinical pregnancy was defined as a positive plasma β-hCG concentration and by the ultrasound detection of a fetal heartbeat after 5 weeks. The implantation rate was defined as the number of gestation sacs on ultrasound as a percentage of the embryos transferred. Live birth was defined as a birth in which one or more fetuses were live-born. Early miscarriage was defined as a clinical pregnancy that was spontaneously miscarried before week 12 of the pregnancy.

R Programming Language was used for data analysis. Pearson’s correlation test, the chi-square test, and Fisher’s exact test were used to assess differences among groups with regard to various rates of development. A value of *P* < 0.05 was considered statistically significant.

## Results

In the present study, 1239 single blastomeres from 329 cycles were assessed for chromosomal aneuploidy. The mean maternal age was 30.8 ± 4.2 years. As shown in Table 1, a total of 94.3% (1168/1239) of the embryos yielded informative results, while 5.7% (71/1239) of embryos failed to produce a result. Among the samples, the overall embryo euploid rate was 21.9% (256/1168), and 78.1% (912/1168) of the embryos were aneuploid. In total, 225 embryos were transferred in 169 cycles, and the remaining 159 cycles were canceled due to a lack of euploid embryos. Single embryo transfer was performed in 113 cycles, and two embryos were transferred in 56 cycles, resulting in a clinical pregnancy rate of 49.1% (83/169). Although the clinical pregnancy and live birth rates of double embryos transfer were higher than single embryo transfer (58.9% vs. 44.2%, *P* = 0.05 and 57.1% vs. 42.5%, *P* = 0.05, respectively), there was no difference of implantation rate between double embryos transfer and single embryo transfer (44.2% vs. 44.6, *P* > 0.05). Two pregnancies ended in early miscarriage (early miscarriage rate 1.2%), and one patient underwent late miscarriage. The live birth rate was 47.3% (80/169). The implantation rate was calculated to be 44.4% (100/225).

**TABLE 1:** Clinical characteristics and outcomes of all the patients for day-3 NGS-PGT.

Variable	Patients
Maternal age (y)	30.8 (4.2)
BMI (kg/m^2^)	22.0 (2.4)
FSH (mIU/ml)	6.5 (2.3)
LH (mIU/ml)	5.3 (3.5)
E_2_ (pg/ml)	93.2 (56.8)
AMH (ng/mL)	3.5 (2.1)
Whole genome amplification rate (%)	94.3 (1168/1239)
Euploid embryos (%)	21.9 (256/1168)
Biopsied cycles	329
Transferred cycles (%)	51.4% (169/329)
Cycles of clinical pregnancy (%)	49.1% (83/169)
Cycles of live birth (%)	47.3% (80/169)
Cycles of early miscarriage (%)	1.2% (2/169)
Embryos transferred	225
Embryos implanted (%)	44.4% (100/225)

*Data are presented as mean (SD) or % (n).*

No significant differences were observed in the percentage of informative results according to the type of structural rearrangement. The percentage of normal/balanced embryos with reciprocal translocations (16.1%) was significantly lower than that of embryos with Robertsonian translocations (28.0%, *P* < 0.01) and inversions (28.0%, *P* < 0.01) (Table 2). Totally, 1224 embryos from reciprocal translocations, Robertsonian translocations, and inversions were included, and 252 euploid embryos were identified from 1154 informative results. The incidence of other types of chromosome abnormalities (insertion, fragmental duplication, and microdeletion) was not calculated separately because of the limited sample size in the present study. Three patients with fragmental duplications or microdeletions underwent 5 NGS-PGT cycles. The segmental sizes varied from 4.6 to 16.0 Mb. The patient with karyotype 46,XX,del(21) was verified to have a 4.6 Mb deletion in 21q22.3 by SNP array. Although only one embryo developed since the patient suffered from premature ovarian insufficiency, maternal microdeletion was successfully detected in the unique embryo (Supplementary Figure 1), suggesting that NGS-PGT is capable of detecting segmental aneuploidy with less than 5 Mb in blastomeres from day-3 embryos.

**TABLE 2:** Incidence of chromosomal abnormalities according to type of rearrangement.

	RecT	RobT	Inv	No CR	*P-*value
Patients	154	62	29	36	N/A
Embryos biopsied	670	291	124	139	N/A
Information results (%)	93.9(629/670)	94.5(275/291)	95.2(118/124)	95.0(132/139)	0.99
Euploid embryos (%)	16.1(101/629)^a,b,c^	28.0(77/275)^a^	28.0(33/118)^b^	31.1(41/132)^c^	< 0.01
Type I (%)	29.6(186/629)^a,b,c^	15.3(42/275)^a,d^	8.5(10/118)^b,e^	0.8(1/132)^c,d,e^	< 0.01
Type II (%)	11.6(73/629)^a,b,c^	23.3(64/275)^a,d^	30.5(36/118)^b,e^	49.2(65/132)^c,d,e^	< 0.01
Type III (%)	15.7(99/629)^a^	13.5(37/275) ^b^	9.3(11/118)^c^	0(0/132)^a,b,c^	< 0.01
Type IV (%)	27.0(170/629)	20(55/275)	23.7(28/118)	18.9(25/132)	0.20
Type I + Type III (%)	45.3(285/629)^a,b,c^	28.7(79/275)^a,d,e^	17.8(21/118)^b,d,f^	0.8(1/132)^c,e,f^	< 0.01
Type II + Type III (%)	27.3(172/629)^a,b,c^	36.7(101/275)^a,d^	39.8(47/118)^b^	49.2(65/132)^c,d^	< 0.01

*Data are presented as % (n). Embryos from patients with insertion, fragmental duplication and microdeletion were not included. RecT, reciprocal translocations; RobT, Robertsonian translocations; Inv, inversions; No CR, no chromosome rearrangement; Type I, abnormalities related to parental rearrangement only (%); Type II, de novo abnormalities only (%); Type III, abnormalities related to parental rearrangement and de novo abnormalities (%); Type IV, multiple aneuploidy of chromosome; Type I + Type III, embryos with abnormalities related to parental arrangement; Type II + Type III, embryos with de novo abnormalities. ^a–f^P < 0.05, for Fisher’s exact test/chi-square test between columns in the same row.*

Here, we divided the chromosomal abnormalities into five different types to analyze their distributions in embryos from patients with different types of rearrangements. Type I: embryos with abnormalities related to parental rearrangement only; Type II: embryos with *de novo* abnormalities only; Type III: embryos with both parental-related and *de novo* abnormalities; Type IV: multiple aneuploidy of chromosome (MAC); and Type V: normal/balanced embryos. Unsurprisingly, the incidence of parental rearrangement-related abnormalities (Type I + Type III) for reciprocal translocations (45.3%) was significantly higher than that for Robertsonian translocations (28.7%, *P* < 0.01) and inversions (17.8%, *P* < 0.01). However, we observed higher percentages of embryos with *de novo* abnormalities only (Type II) for Robertsonian translocations (23.3%, *P* < 0.01) and inversions (30.5%, *P* < 0.01) than for reciprocal translocations (11.6%). Moreover, the percentages of embryos with *de novo* abnormalities (Type II + Type III) for Robertsonian translocations (36.7%, *P* < 0.01) and inversions (39.8%, *P* = 0.02) were also higher than those for reciprocal translocations (27.3%). Embryos from patients without chromosomal rearrangements (Y chromosome microdeletions and sex chromosome abnormalities) also presented a high percentage of *de novo* abnormalities (49.2%) (Table 2) with the highest incidence of aneuploidy of chromosome 21. The most common *de novo* abnormalities for all the embryos were aneuploidy of chromosome 22, chromosome 19, followed by chromosome 21 and chromosome 16. Our results further confirmed that *de novo* abnormalities are common in cleavage-stage embryos.

Given the low percentage of normal/balanced embryos for reciprocal translocation, only 41.5% of reciprocal translocation cycles had euploid embryos for transfer, which was significantly lower than that for Robertsonian translocations (61.0%, *P* < 0.01) and inversions (65.7%, *P* < 0.01) (Table 3). However, when a euploid embryo was transferred, there was no significant difference in clinical pregnancy, early miscarriage, embryo implantation, and live birth rates among the rearrangement types (Table 3). In one special case, both the mother and the father were reciprocal translocation carriers, with karyotypes of 46, XX, t(2;8)(q24;p22) and 46, XY, t(11;22)(q23;q11.2), respectively. Theoretically, the couple had only a 1/81 opportunity to obtain euploid embryos. A day-3 PGT strategy was suggested to the couple to have more embryos available for biopsy. Finally, 4 embryos were subjected to NGS-PGT on day 3, and one showed normal/balanced results (Supplementary Figure 2). The euploid embryo was transferred on day 4, resulting in a healthy live birth.

**TABLE 3:** Clinical outcome according to type of rearrangement.

	RecT	RobT	Inv	No CR	*P*-value
Patients	154	62	29	36	N/A
Maternal age (y)	29.8(3.5)	30.3(4.2)	30.0(3.3)	29.5(4.3)	0.67
BMI (kg/m^2^)	22.0(2.7)	22.5(2.2)	21.3(2.4)	22.2(2.4)	0.13
FSH (mIU/ml)	6.4(2.3)	6.3(2.7)	5.9(2.1)	6.9(2.7)	0.42
LH (mIU/ml)	5.7(4.2)	5.3(3.8)	4.5(2.6)	10.8(33.5)	0.08
E_2_ (pg/ml)	100.2(60.0)	85.8(55.5)	91.1(52.2)	89.9(47.2)	0.29
Biopsied cycles	171	77	35	40	N/A
Transferred cycles (%)	41.5 (71/171)^a,b,c^	61.0 (47/77)^a^	65.7 (23/35)^b^	65.0 (26/40)^c^	< 0.01
Embryos biopsied	670	291	124	139	N/A
Euploid embryos (%)	16.1 (101/629)^a,b,c^	28 (77/275)^a^	28.0 (33/118)^b^	31.1 (41/132)^c^	< 0.01
Embryo transferred per cycle	1.3(0.5)	1.5(0.5)	1.2(0.4)	1.3(0.5)	0.10
Implantation rate (%)	51.2(47/92)	37.7 (26/69)	39.3 (11/28)	48.5 (16/33)	0.33
Clinical pregnancy rate (%)	57.8 (41/71)	42.6 (20/47)	39.1 (9/23)	50.0 (13/26)	0.28
Live birth rate (%)	56.3 (40/71)	40.4 (19/47)	39.1 (9/23)	50.0 (13/26)	0.28
Early miscarriage rate (%)	2.4 (1/41)	5.0 (1/20)	0.0 (0/9)	0.0 (0/13)	0.77

*Data are presented as mean (SD) or % (n). Embryos from patients with insertion, fragmental duplication and microdeletion were not included. RecT, reciprocal translocations; RobT, Robertsonian translocations; Inv, inversions; No CR, no chromosome rearrangement. ^a–c^P < 0.05, for Fisher’s exact test/chi-square test between columns in the same row.*

In cycles not suitable for fresh embryo transfer [risk of ovarian hyperstimulation syndrome (OHSS), poor endometrium, infection, etc.], good-quality embryos were cryopreserved, and PGT was performed in frozen embryo transfer (FET) cycles. Clinical outcomes did not show significant differences between fresh and FET cycles (Table 4). Clinical pregnancy, early miscarriage and live birth rate were similar in fresh and FET cycles (Table 4). FET cycles presented a higher implantation rate than fresh cycles (49.1% *vs*. 39.4%, *P* = 0.18), but the difference was not statistically significant (Table 4). The basal FSH levels were lower in the FET group due to the inclusion of patients with high-risk OHSS, but the maternal age and BMI were not different between fresh and FET cycles (Table 4). Our results suggest that day-3 PGT performed in FET cycles would be a feasible alternative to that for fresh cycles.

**TABLE 4:** Clinical outcomes between Fresh and FET cycles.

	Fresh cycles	FET cycles	*P-*value
Maternal age (y)	29.7(3.6)	29.4(3.96)	0.13
BMI (kg/m^2^)	21.9(2.5)	21.8(2.4)	0.77
FSH (mIU/ml)	6.5(2.3)	6.0(2.2)	0.01
LH (mIU/ml)	5.2(3.5)	6.2(13.3)	0.25
E_2_ (pg/ml)	98.8(59.9)	108.6(132.5)	0.26
Biopsied cycles	172	157	0.13
Transferred cycles (%)	47.1 (81/172)	56.1 (88/157)	0.13
Embryos biopsied	629	610	N/A
Euploid embryos (%)	22.3 (131/587)	21.5 (125/581)	0.80
Embryo transferred per cycle	1.3(0.5)	1.3(0.5)	0.70
Implantation rate (%)	39.4 (43/109)	49.1 (57/116)	0.18
Clinical pregnancy rate (%)	45.7 (37/81)	52.3 (46/88)	0.48
Live birth rate (%)	45.7 (37/81)	48.9 (43/88)	0.79
Early miscarriage rate (%)	0.0 (0/37)	4.3 (2/46)	0.57

*Data are presented as mean (SD) or % (n).*

Although most of the patients in the present study suffered from chromosomal rearrangement, the euploid rates in patients < 35 and ≥ 35 years of age were not significantly different. In other words, in contrast to patients with normal karyotypes, the aneuploidy rate of cleavage-stage embryos from structural arrangement carriers did not increase significantly with age. We also did not observe a significant difference in clinical outcomes between patients < 35 and ≥ 35 years of age (Table 5). Age does not appear to be a crucial factor in structural arrangement carriers who undergo PGT-SR as long as they have enough good-quality embryos. We also compared the data from the male carrier and female carrier for the chromosomal rearrangements and found that there is no significant difference between the two groups in terms of euploid embryo rates and the clinical outcomes (Supplementary Table 1).

**TABLE 5:** Clinical outcomes between patients < 35 and ≥ 35 years old.

	Age < 35	Age ≥ 35	*P*-value
Maternal age (y)	28.7(2.7)	36.9(2.4)	< 0.01
BMI (kg/m^2^)	21.8(2.5)	22.5(2.1)	0.04
FSH (mIU/ml)	6.2(2.2)	6.3(2.3)	0.63
LH (mIU/ml)	5.4(3.5)	7.7(27.1)	0.52
E_2_ (pg/ml)	102.9(106.1)	109.4(58.1)	0.45
Biopsied cycles	284	45	N/A
Transferred cycles (%)	52.4(149/284)	44.4(20/45)	0.39
Embryos biopsied	1088	151	N/A
Euploid embryos (%)	22.3 (228/1023)	19.3 (28/145)	0.59
Embryo transferred per cycle	1.3(0.5)	1.3(0.5)	0.96
Implantation rate (%)	44.2 (88/199)	46.2 (12/26)	1.00
Clinical pregnancy rate (%)	49.0 (73/149)	50.0 (10/20)	1.00
Live birth rate (%)	47.0 (70/149)	50.0 (10/20)	0.98
Early miscarriage rate (%)	2.7 (2/73)	0.0 (0/10)	1.00

*Data are presented as mean (SD) or % (n).*

In the present study, 1–3 embryos that had a low chance of yielding good-quality blastocysts were biopsied in nearly half of the cycles (Supplementary Figure 3). Although the cancelation rate (lack of euploid embryos for transfer) decreased as the number of embryos biopsied increased, the clinical pregnancy and live birth rates were not significantly different between cycles with 1–3 and > 3 embryos (47.1% vs. 50% and 45% vs. 48.3%, *P* = 0.85 and *P* = 0.83, respectively) biopsied. Similarly, the implantation rates between the two groups were the same (44.4% vs. 44.4%, *P* = 0.88) when a euploid embryo was transferred. The clinical outcomes according to the number of embryos biopsied are listed in Table 6.

**TABLE 6:** Clinical outcomes of cycles with different numbers of embryos for biopsy.

	Transfer cycles/biopsy cycles (%)	Implantation rate (%)	Clinical pregnancy rate (%)	Live birth rate (%)
1 embryo biopsied	16.7(6/36)^*a,b,c,d,e,f*^	16.7(1/6)	16.7(1/6)	16.7(1/6)
2 embryos biopsied	29.0(18/62)^*g,h,i,j,k*^	54.5(12/22)	61.1(11/18)	55.6(10/18)
3 embryos biopsied	45.0(27/60)^*a,l,m,n,o*^	42.9(15/35)	44.4(12/27)	44.4(12/27)
4 embryos biopsied	59.7(37/62) ^*b,g*^	46.8(22/47)	43.2(16/37)	40.5(15/37)
5 embryos biopsied	71.2(37/52)^*c,h,l*^	46.0(23/50)	51.4(19/37)	51.4(19/37)
6 embryos biopsied	79.3(23/29)^*d,i,m*^	41.9(13/31)	52.2(12/23)	52.2(12/23)
7 embryos biopsied	68.8(11/16)^*e,j,n*^	31.6(6/19)	54.5(6/11)	45.5(5/11)
≥8 embryos biopsied	83.3(10/12)^*f,k,o*^	53.3(8/15)	60.0(6/10)	60.0(6/10)
*P*-value	< 0.01	0.68	0.74	0.73

*Note: Data are presented as % (n). ^a–o^P < 0.05, for chi-square test between rows in the same column.*

## Discussion

The present study describes the successful implementation of MALBAC-NGS analysis on day-3 embryos for PGT by detecting aneuploidy and segmental aneuploidy in patients with structural chromosomal rearrangements, Y chromosome microdeletions or sex chromosome abnormalities. Our study yielded 49.1% clinical pregnancy, 47.3% live birth, 1.2% miscarriage, and 44.4% implantation rates. Double embryos transfer revealed higher clinical pregnancy and live birth rates compared with single embryo transfer, but the implantation rates were similar. Our results confirmed that NGS for PGT on day-3 embryos is an effective clinical application, especially for patients who have only a few embryos available for biopsy.

Several CCS techniques, including aCGH, SNP, and NGS, have been applied for PGT at the blastocyst stage. However, only a few studies have reported CCS-PGT for day-3 embryos (Keltz et al., 2013; Lukaszuk et al., 2015; Dimitriadou et al., 2017; Mateu-Brull et al., 2019). Compared with single-blastomere biopsies from cleavage-stage embryos, 5–10 cells can be biopsied from blastocysts, making genetic diagnosis more reliable. The sensitivity and specificity of PGT results were greatly dependent on the WGA used. Currently, the main WGA methods used for PGT include degenerate oligonucleotide-primed polymerase chain reaction (DOP-PCR) (Telenius et al., 1992), multiple-displacement amplification (MDA) (Dean et al., 2002), and MALBAC (Zong et al., 2012; Blanshard et al., 2018). By introducing a quasilinear amplification step, MALBAC could significantly reduce the amplification bias and has the further advantage of accurately detecting copy number variations (CNVs) (Huang et al., 2015). It has been demonstrated that the CNV detection rates in the single-cell samples are higher for MALBAC than for MDA; the coefficient of variation in the CNV detection for MALBAC was significantly superior to that of MDA (0.15 vs. 0.37) (Liu et al., 2018).

Generally, blastocyst biopsy combined with FET is performed for PGT. However, the *in vitro* environment is likely inferior to that *in vivo*, and there is a risk of losing some embryos during extended culture (Martins et al., 2017). Thus, patients with insufficient ovarian function due to advanced maternal age, ovarian surgery, and autoimmune diseases may have difficulties developing good-quality blastocysts for biopsy. Moreover, embryos developing to blastocysts are not a guarantee of chromosomal normality. Previous NGS-PGT studies revealed that the euploidy rates of blastocysts from patients carrying reciprocal translocation were approximately 20–30% (Cai et al., 2019; Zhang et al., 2019). Some embryos failing to blastulate in culture may be euploid and have the chance to implant successfully if transferred at an earlier stage (Martins et al., 2017). Although trophectoderm biopsy is believed to have a smaller detrimental effect on embryo viability compared to biopsy on day 3, the clinical outcomes after blastocyst biopsy depend on the biopsy technique (Rubino et al., 2020). The implantation rates of blastocysts following trophectoderm biopsy vary from 30 to 60% (Christodoulou et al., 2017; Mateu-Brull et al., 2019; Rubino et al., 2020). Our results demonstrated an implantation rate of 44.4% after day-3 embryo biopsy, which was significantly higher than the implantation rate after ICSI treatment (30.8%) in our center during the same period. Other studies also showed satisfying clinical outcomes after CCS-PGT with day-3 embryos (Keltz et al., 2013; Lukaszuk et al., 2015). It has been suggested that trophectoderm biopsy reduces the risk of mosaicism, but analysis of the NGS results from day-3 and day-5 embryo biopsies showed a concordance rate per embryo diagnosis of 85.6% for euploid and aneuploid embryos (Liñán and Lawrenz, 2018). Our day-3 embryo NGS-PGT strategy may help patients with poor blastocyst formation have a better prognosis.

Reciprocal translocations, Robertsonian translocations and inversions are the most common structural chromosomal rearrangements, with an increased risk of producing gametes related to the arrangements. It has been proposed that ICE in carriers of structural rearrangements might be responsible for chromosomal abnormalities not related to rearrangements. Chromosomes involved in rearrangements might influence the segregation of other structurally normal chromosomes during meiosis, leading to an increase in aneuploidy gametes (Wang et al., 2019). Reciprocal translocations, Robertsonian translocations, and inversions are the most common structural chromosomal rearrangements, with an increased risk of producing gametes related to the arrangements. It has been proposed that ICE in carriers of structural rearrangements might be responsible for chromosomal abnormalities not related to rearrangements. Chromosomes involved in rearrangements might influence the segregation of other structurally normal chromosomes during meiosis, leading to an increase in aneuploidy gametes (Wang et al., 2019). However, some studies suggested that ICE was of possible mitotic rather than a meiotic origin (Alfarawati et al., 2012; Mateu-Brull et al., 2019). Studies in spermatozoa (Anton et al., 2011; Godo et al., 2015), oocytes (Durban et al., 2001; Alfarawati et al., 2012), and embryos (Gianaroli et al., 2002; Alfarawati et al., 2012) have indicated that Robertsonian translocations appear to be more prone to have ICEs than reciprocal translocations. Our results illustrated that the proportion of embryos with *de novo* chromosomal abnormalities was significantly higher for Robertsonian translocations and inversions than for reciprocal translocation, which is in accordance with a previous study (Mateu-Brull et al., 2019). All these data suggest that the incidence of *de novo* aneuploidy varies among different types of structural chromosomal rearrangements, and further studies are needed to elucidate whether ICEs truly exist and to investigate the underlying mechanisms. In this study, embryos from patients without chromosomal rearrangements also presented with a high percentage of aneuploidy, which is possibly related to the fact that this group was composed largely of patients with Y chromosome microdeletions. Latest studies have suggested that sperm aneuploidy is associated with semen quality (Sachdeva et al., 2020), and high rates of aneuploidy and mosaicism have been detected in embryos from patients with severe male infertility (Kahraman et al., 2020).

Our results did not demonstrate any significant difference in clinical outcomes between fresh and FET cycles, indicating that NGS-PGT with frozen-thawed day-3 embryos would not necessarily decrease implantation potential. PGT accompanied with FET would be a feasible option when embryo transfer cannot be performed in the oocyte retrieval cycle. We also did not observe a difference in clinical outcomes between patients < 35 and ≥ 35 years of age, suggesting that euploid embryos can be implanted with similar efficiency regardless of the age of the mother. The PGT-A study also demonstrated that once a single euploid embryo is transferred, high levels of implantation and live birth success can be attained independent of patient age (Anderson et al., 2020).

## Conclusion

In conclusion, our study verified the clinical validity of single blastomere NGS-PGT for chromosomal disorder patients for selecting a euploid embryo for transfer. Furthermore, we demonstrated that PGT based on MALBAC-NGS is applicable not only for trophectoderm biopsies but also for single cells. A prospective PGT-A trial performed with day-3 embryos using NGS also exhibited excellent implantation and clinical pregnancy rates and a low miscarriage rate (Lukaszuk et al., 2015). In contrast to this trial, we recruited a large number of chromosomal disorder patients. Moreover, the novel tool is sufficiently rapid to allow embryo transfer the day after biopsy. Future randomized clinical trials are required to validate the present results, and these data would be a strong argument for a wider application of the NGS-PGT strategy with day-3 embryos not only for chromosomal disorder patients carrying balanced structural chromosomal rearrangement but also for normal karyotype patients to select euploid embryos with the best implantation potential.

## Data Availability Statement

The datasets presented in this study can be found in online repositories: CNGB Sequence Archive (CNSA) of China National GeneBank DataBase (CNGBdb). The data can be accessed via the following link: http://db.cngb.org/cnsa/project/CNP0001561/reviewlink/.

## Ethics Statement

The studies involving human participants were reviewed and approved by the Medical Ethics Committee of the Women’s Hospital, Zhejiang University School of Medicine (IRB-20200242-R). Written informed consent for participation was not required for this study in accordance with the national legislation and the institutional requirements.

## Author Contributions

YY and FJ designed the study. JM performed the data analysis. YY wrote the manuscript. LC participated in the data analysis and visualization. SL critically reviewed the manuscript. All authors reviewed and approved the final manuscript.

## Conflict of Interest

JM and SL were employed by the company Yikon Genomics Co. Ltd. The remaining authors declare that the research was conducted in the absence of any commercial or financial relationships that could be construed as a potential conflict of interest.

## Supplementary Material

Supplementary Figure 1 Embryo CNV pattern for a patient with a 4.6 Mb loss in 21q22.3. The embryo 15A1704100201 was detected *de novo* loss at chr 14 and genetic loss at 21q22.3 for 4.6 Mb at the depth of 0.04 ×. The X-axis represents 24 chromosomes, and the number of Y-axis means the copy number of the chromosomes.

Supplementary Figure 2 Embryo CNV patterns for a female and male couple who were both reciprocal translocation carriers with 46,XX,t(2;8)(q24;p22) and 46,XY,t(11;22)(q23;q11.2) karyotypes, respectively. Chromosome aneuploidies were detected except the embryo 15A170622AP01CB03. The X-axis represents 24 chromosomes, and the number of Y-axis means the copy number of the chromosomes.

Supplementary Figure 3 Proportional distribution of cycles with different numbers of embryos biopsied.
