# Supplementary figures and images for: A Rapid NGS-Based Preimplantation Genetic Testing for Chromosomal Abnormalities in Day-3 Blastomere Biopsy Allows Embryo Transfer Within the Same Treatment Cycle
Source: Front Genet. 2021 Feb 26;12:636370. doi: 10.3389/fgene.2021.636370 (PMC7952972; doi:10.3389/fgene.2021.636370)

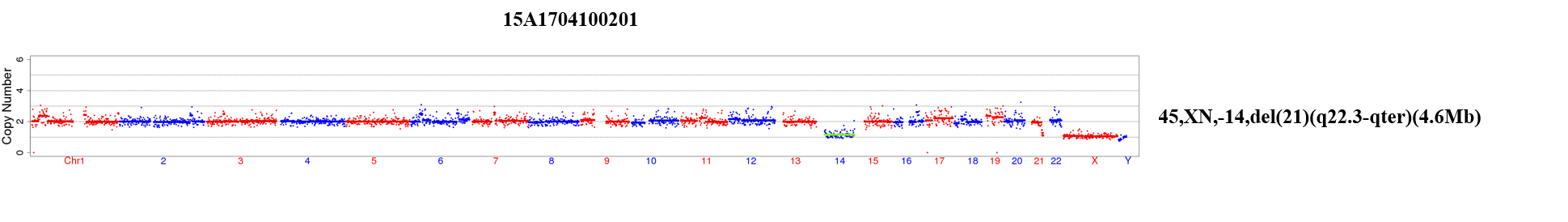

Supplement: Supplementary Figure 1 — Embryo CNV pattern for a patient with a 4.6 Mb loss in 21q22.3. The embryo 15A1704100201 was detected de novo loss at chr 14 and genetic loss at 21q22.3 for 4.6 Mb at the depth of 0.04 ×. The X-axis represents 24 chromosomes, and the number of Y-axis means the copy number of the chromosomes. [file Image_1.TIF]

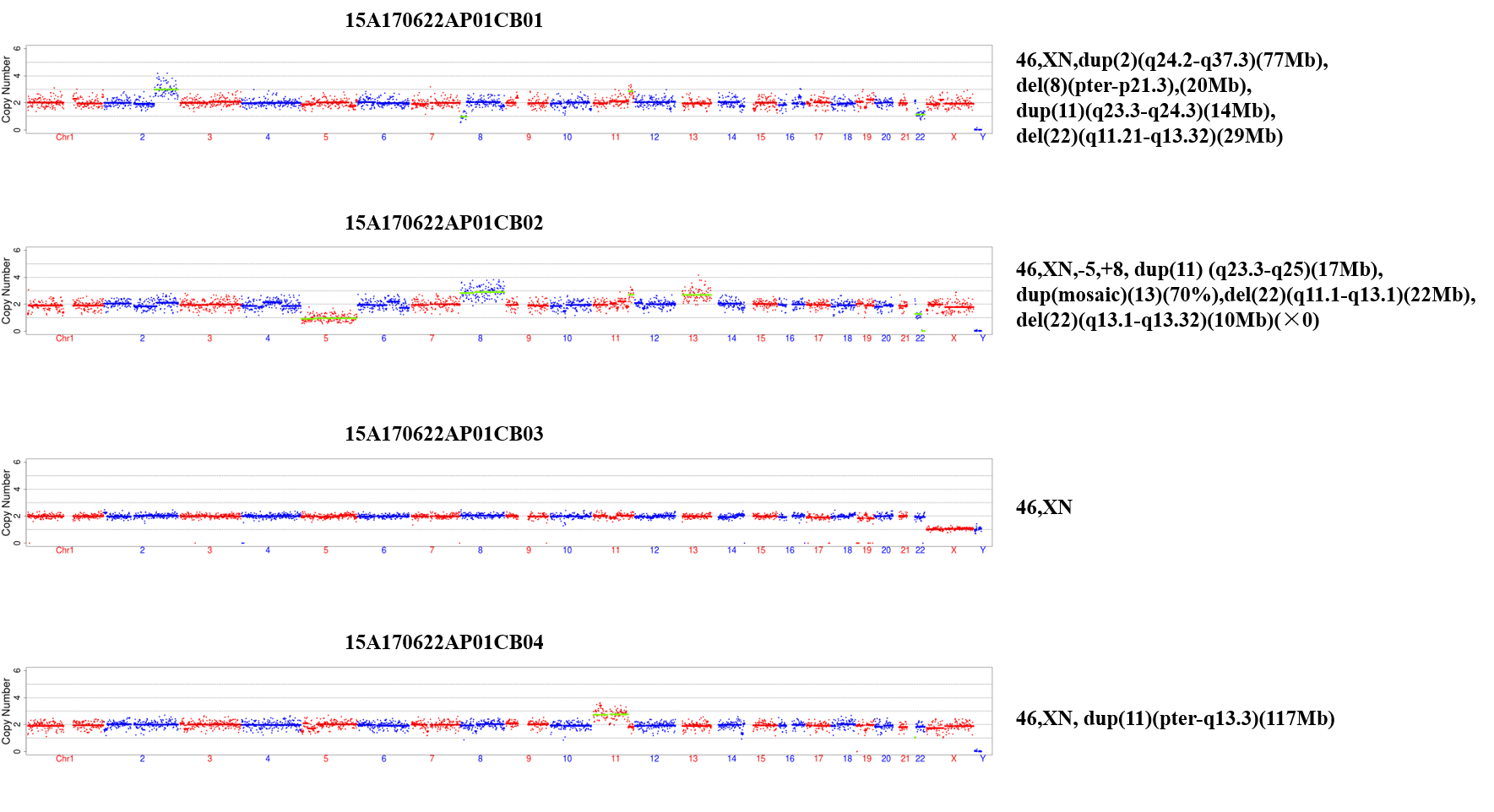

Supplement: Supplementary Figure 2 — Embryo CNV patterns for a female and male couple who were both reciprocal translocation carriers with 46,XX,t(2;8)(q24;p22) and 46,XY,t(11;22)(q23;q11.2) karyotypes, respectively. Chromosome aneuploidies were detected except the embryo 15A170622AP01CB03. The X-axis represents 24 chromosomes, and the number of Y-axis means the copy number of the chromosomes. [file Image_2.TIF]

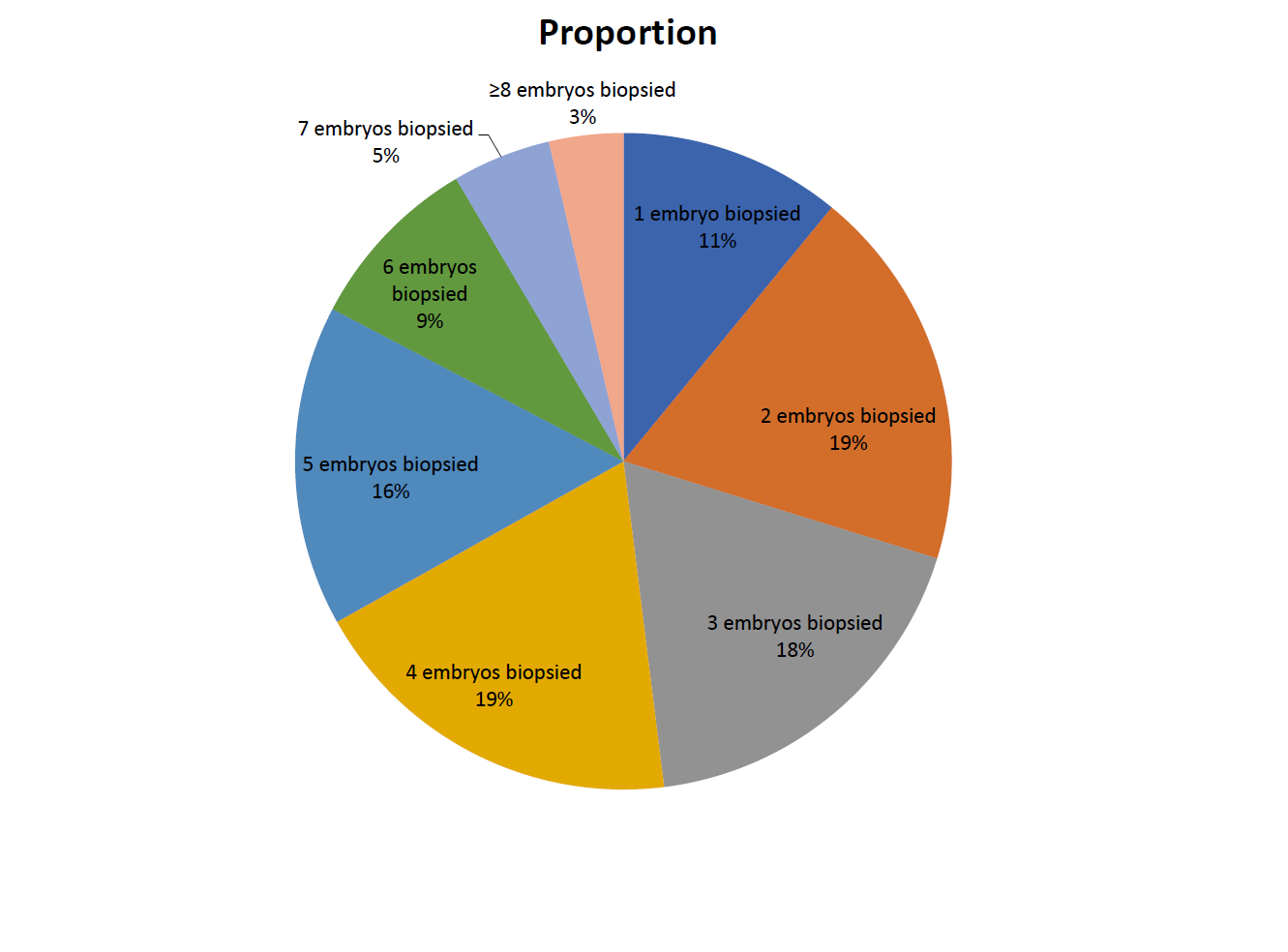

Supplement: Supplementary Figure 3 — Proportional distribution of cycles with different numbers of embryos biopsied. [file Image_3.TIF]
