# Supplementary material for: A Rapid NGS-Based Preimplantation Genetic Testing for Chromosomal Abnormalities in Day-3 Blastomere Biopsy Allows Embryo Transfer Within the Same Treatment Cycle
Source: Front Genet. 2021 Feb 26;12:636370. doi: 10.3389/fgene.2021.636370 (PMC7952972; doi:10.3389/fgene.2021.636370)
Supplement: Supplementary file 4 [file Table_1.DOCX]

Supplementary Table 1

**Clinical outcomes between** **female structure rearrangement carriers and male carriers.**

|  | Female carrier | Male carrier | *P* value |
| --- | --- | --- | --- |
| Patients | 103 | 130 | N/A |
| Maternal Age (y) | 29.8 (3.6) | 30.1(3.8) | 0.44 |
| BMI(Kg/m^2^) | 22.0(2.2) | 22.2(2.8) | 0.65 |
| FSH (mIU/ml) | 6.1(2.3) | 6.2(2.2) | 0.61 |
| LH (mIU/ml) | 5.0(3.4) | 5.8(4.4) | 0.16 |
| E_2_ (pg/ml) | 97.4(65.7) | 97.4(56.0) | 0.99 |
| Biopsied cycles | 142 | 149 | N/A |
| Transferred cycles (%) | 45.0(64/142) | 54.4(20/45) | 1.00 |
| Embryos biopsied | 521 | 587 | N/A |
| Euploid embryos (%) | 18.2 (95/521) | 21.0 (123/587) | 0.39 |
| Embryo transferred per cycle | 1.3(0.5) | 1.4(0.5) |  |
| Implantation rate (%) | 42.9(36/84) | 44.1 (49/111) | 1.00 |
| Clinical pregnancy rate (%) | 49.0 (33/64) | 46.9 (38/81) | 0.86 |
| Live birth rate (%) | 47.0 (31/64) | 50.0 (37/81) | 0.96 |
| Early miscarriage rate (%) | 3.0(1/33) | 2.6 (1/38) | 1.00 |
